# Validation of a Mobile Health Device for Neonatal Jaundice Screening: A Cross-Sectional Study in a Resource-Limited Setting in Mexico

**DOI:** 10.1177/30502225251320544

**Published:** 2025-04-22

**Authors:** Gabriela Jiménez-Díaz, Lobke M. Gierman, Martina Keitsch, Jesús Elizarrarás-Rivas, Rey Manuel Silva- Méndez, Jennifer J. Infanti, Anna Marcuzzi

**Affiliations:** 1 NTNU-Norwegian University of Science and Technology, Trondheim, Norway; 2 Picterus AS, Trondheim, Norway; 3 Mexican Institute of Social Security, IMSS, Oaxaca, Mexico; 4 Universidad Autónoma Benito Juárez de Oaxaca, Oaxaca City, México; 5 St. Olavs Hospital, Trondheim, Norway

**Keywords:** neonatal jaundice, neonatal hyperbilirubinemia, detection, screening, mobile health, resource-limited settings, low-resource settings

## Abstract

**Objective:**

To evaluate the performance of Picterus Jaundice Pro (Picterus JP), a mobile health device for neonatal jaundice (NNJ) screening, in Mexican newborns.

**Methodology:**

A cross-sectional study was conducted from January 2023 to June 2024 at a hospital in a resource-limited setting in Mexico. The main outcomes were Picterus JP measurements and total serum bilirubin (TSB) levels.

**Results:**

A total of 177 term newborns, aged 1 to 14 days, were enrolled. Picterus JP showed a significant positive Spearman’s rho correlation with TSB (ρ = .68), sensitivity of 85.7% and specificity of 80.1% to detect TSB ≥ 250 using Picterus cut off value of 202 µmol/L. However, it tended to underestimate higher bilirubin levels.

**Conclusion:**

Picterus JP shows potential to be a useful tool for NNJ screening, particularly in resource-limited areas. Further validation across more diverse populations and clinical environments, alongside accuracy improvements, is necessary to enhance its utility and support wider implementation.

Registered in ClinicalTrials.gov (https://clinicaltrials.gov/study/NCT06276582)

## Introduction

Neonatal jaundice (NNJ), a clinical sign of hyperbilirubinemia, is a global health issue affecting over half of newborns within the first 48 to 72 hours after birth.^1^ If undetected or untreated, it can cause permanent brain damage, leading to long-term disabilities or even death.^2-4^ While most NNJ cases are harmless and self-limiting, around 1 million of newborns annually develop severe NNJ, requiring close monitoring and treatment.^5^

Accurate detection of NNJ remains a significant challenge, especially in low- and middle-income countries (LMICs), other resource-limited settings, and non-hospital locations. The gold standard for diagnosing NNJ, the total serum bilirubin (TSB) test, requires laboratory facilities and is invasive. Screening devices like transcutaneous bilirubinometers (TcB), while offering high sensitivity for detecting hyperbilirubinemia (74%-100%),^6^ are often inaccessible due to their high cost, typically ranging from $3,000 to $6,000 USD.^7-11^ Consequently, detection frequently relies on visual assessment (VA), which is inaccurate and unreliable, contributing to a higher prevalence of severe NNJ in these settings compared to high-income countries.^1,12,13^

The widespread adoption of smartphones has paved the way for mobile health (mHealth) tools, offering non-invasive and potentially affordable alternatives for NNJ screening. Several smartphone-based devices have shown promise in early identification of severe cases.^14-19^ Picterus Jaundice Pro (Picterus JP) is a mobile device developed to support NNJ screening and follow-up. It includes a smartphone app for capturing and uploading images, a calibration card for adjusting light conditions and camera performance, and a server for image storage and analysis. The device uses optical measurements of bilirubin levels from images of a newborn’s chest. Picterus JP has been validated in Norway and pilot studies in Mexico and the Philippines, demonstrating a positive correlation with TSB levels and superior accuracy compared to VA.^20-23^

Despite significant advancements, smartphone-based tools like Picterus JP still face limitations, including sensitivity to varying lighting conditions and skin pigmentation. These challenges have been addressed through ongoing improvements to the app algorithm and the calibration card. Unlike prior validations conducted in Mexico, Norway, and the Philippines, this study evaluates an updated Picterus JP with a newer version of the calibration card in a distinct resource-limited setting in Mexico.

With nearly 130 million inhabitants and a diverse healthcare system,^24,25^ Mexico presents a unique context for this evaluation. This study focuses on the device’s accuracy as a screening tool for NNJ in newborns from a resource-limited setting, providing valuable insights into its practical implementation and potential to improve neonatal care in similar global contexts.

## Material and Methods

### Study Setting and Design

A cross-sectional study was conducted at Hospital General de Zona 1 (HGZ) in Oaxaca de Juarez, Mexico, from January 2023 to June 2024. Oaxaca, known for its ethnic, linguistic, and cultural diversity,^26^ is one of the country’s poorer states. This is a second-level hospital under the Instituto Mexicano del Seguro Social (IMSS), providing specialized care such as general surgery, obstetric services ad neonatal care, handling approximately 2,000 births annually.

### Participants and Sample Size

Participants were recruited from the maternity ward and emergency room. Inclusion criteria were term newborns (gestational age of 37 weeks or more), aged 1 to 14 days, with a birth weight of ≥2500 grams, and who required a blood test for any medical reason to minimize unnecessary punctures. Newborns with congenital diseases, those transferred to paediatric wards for reasons unrelated to NNJ treatment, or those who had undergone phototherapy for NNJ were excluded.

The sample size was determined based on sensitivity and specificity analysis, assuming a severe jaundice (TSB ≥ 250 µmol/L; 14.6 mg/dL) prevalence of 10%. A sample size of 120-200 newborns was required to achieve a minimum power of 80% for an expected sensitivity and specificity between 80% and 90%.^27^ Thus, the minimum sample size was set at 150 participants.

### Study Procedures

Medical staff from the paediatric ward, trained and supervised through regular visits by the first author, collected the data. After obtaining informed consent, background information was retrieved from medical records, including birth time and date, birth weight, and gestational age. The Fitzpatrick scale was used to assess parental skin types. This classification categorizes adult skin based on its reaction to sun exposure and includes six categories ranging from very light/pale to very dark brown tones.^28^ Newborn skin tones were classified using the Neomar scale, a tool specifically developed for newborns. This scale assesses skin pigmentation in areas such as the chest, areola, and genitals, classifying skin tones into four groups, ranging from white/pink to brown/dark tones.^29^

### Visual Assessment

VA was performed using the Kramer scale, a clinical method for estimating the severity of jaundice in newborns. The scale divides the body into five zones, starting from the head and progressing downward (cephalo-caudal progression), as jaundice spreads with increasing bilirubin levels in the blood. A score from 1 to 5 is assigned based on the lowest zone where yellow discoloration is visible.^30^

### Image Capture

A clinical trial version of Picterus JP (2.7.3, Picterus AS, Norway) was installed on randomly selected smartphones based on their availability at the study site (Samsung A23, iPhone 11, and iPhone 15 Pro) to obtain bilirubin results. During the procedure, a calibration card was placed on the newborn’s chest, and six images were automatically captured – three with flash and three without. These images were stored for later analysis. To ensure unbiased results, medical staff collecting the data were blinded to the bilirubin levels obtained by Picterus JP, preventing any influence on standard neonatal care. A unique measurement ID was recorded to link clinical data with the digital images after the study was completed. Upon completion, bilirubin levels were calculated using the Picterus JP algorithm (Jaundice Image Estimates [JIE], 1.9.14, Picterus AS).

### TSB Measurements

TSB levels were measured with a blood sample obtained by venous puncture on the back side of the newborn’s hand, within one hour of capturing the images. Samples were immediately processed at the hospital laboratory by a vanadate oxidation method using an Atellica® CH analyser (Siemens Healthineers AG, Germany).

### Data Analysis

Descriptive statistics were used to report the participants’ clinical characteristics. Normality of the TSB, Picterus JP, and Kramer scale values was assessed using the Kolmogorov-Smirnov test. Since the data were non-normally distributed, Spearman’s rho correlation coefficient (ρ) was used to determine correlations between Picterus JP, the Kramer scale, and TSB measurements. A Bland-Altman (BA) analysis evaluated the agreement between Picterus JP and TSB, identifying potential biases and outliers. Given the non-normal distribution of the mean difference, both transformed and untransformed datasets were analysed, yielding comparable results. For clearer clinical interpretation, the BA plot with the original, untransformed data is presented. Receiver Operating Characteristic (ROC) curves were generated, and the area under the curve (AUC) was used to evaluate the sensitivity and specificity of detecting severe NNJ at different Picterus JP and Kramer scale cut-off levels. For this study, severe NNJ was defined as TSB ≥ 250 µmol/L (14.6 mg/dL) based on TcB thresholds commonly used to confirm the diagnoses with TSB, as recommended in several clinical guidelines for NNJ.^31-33^ Given that Picterus JP is designed as a screening device, this definition seemed appropriate. Data analysis was conducted using IBM SPSS Statistics version 29.0.1.0 and GraphPad Prism version 10.3.0.

### Ethical Considerations and Consent to Participate

The study was approved by ethical committees in Norway and Mexico: the Regional Committee for Medical and Health Research Ethics (REK), reference number 519379, and the National Committee for Scientific Research, IMSS, reference number R-2022-785-061.

The study objectives were explained to the participants’ parents, who provided written informed consent prior to participation. Confidentiality and anonymity of the participants were ensured. All methods were carried out in accordance with relevant guidelines and regulations.

## Results

A total of 177 newborns were recruited during the study period. Thirteen were excluded due to not meeting the inclusion criteria or technical issues such as poor image quality, leaving 164 participants for analysis. Among these, 28 participants (17%) were classified as having severe NNJ.

The descriptive statistics of the participants’ clinical characteristics, categorized by age at inclusion, are presented in Table 1. Birthweight and gestational age were comparable across the age groups. TSB levels tended to increase after 72 hours post-birth.

**Table 1.** Descriptive Statistics of Clinical Characteristics of Participants, Categorized by Age at Inclusion.

	All ages N = 164		1-3 days n=108		>3 < 7 days n = 46		≥ 7 days n = 10	
Variable	Mean ± SD	Range	Mean ± SD	Range	Mean ± SD	Range	Mean ± SD	Range
Birthweight (grams)	3189 ± 399	2500-4480	3180 ± 405	2500-4480	3196 ± 396	2540-4290	3201 ± 379	2520-3920
Gestational age (weeks)	38.7 ± 1.2	37-42	38.8 ± 1.2	37-42	38.7 ± 1.1	37-41	38.9 ± 1.3	37-41
TSB (µmol/L) (mg/dL)	187.3 ± 80.3 10.9 ± 4.7	58-511 3.4-29.9	158.1 ± 51.9 9.2 ± 3	77-376 4.5-22	245.1 ± 90.4 14.3 ± 5.2	74-511 4.3-29-9	235.4 ± 120.3 13.7 ± 7	58-431 3.4-25.2
Picterus JP (µmol/L) (mg/dL)	172.5 ± 57.1 10.1 ± 3.3	8-319 0.5-18.7	154.8 ± 47.29 ± 2.7	64-290 3.7-17	206.1 ± 57.212 ± 3.3	8-308 0.5-18	209 ± 72.1 12.2 ± 4.2	95-319 5.6-18.7

SD: Standard Deviation; TSB: Total Serum Bilirubin; µmol/L: Micromoles per Litre; mg/dL: Milligrams per Decilitre;.Picterus JP: Picterus Jaundice Pro.

The majority of mothers’ skin types were classified as Fitpatrick scale score III (Table 2). The skin type for fathers was less frequently available, as they are less likely to be present in the hospital. Most newborns’ skin colours were classified as light to medium dark according to the Neomar scale (Table 3).

**Table 2.** Fitzpatrick Scale Scores for Parents’ Skin Type.

	Mother	Father
Score	n (%)	n (%)
II	27 (16.5)	3 (1.8)
III	85 (51.8)	4(2.4)
IV	49 (29.9)	13 (7.9)
V	2 (1.2)	3 (1.8)
Missing	1 (0.6)	141 (86.0)
Total	164 (100)	164 (100)

**Table 3.** Neomar Scale Score for Participants’ Skin Tone.

Score	n	(%)
1	62	37.8
2	54	32.9
3	45	27.4
4	3	1.8
Total	164	100

**Figure 1.**
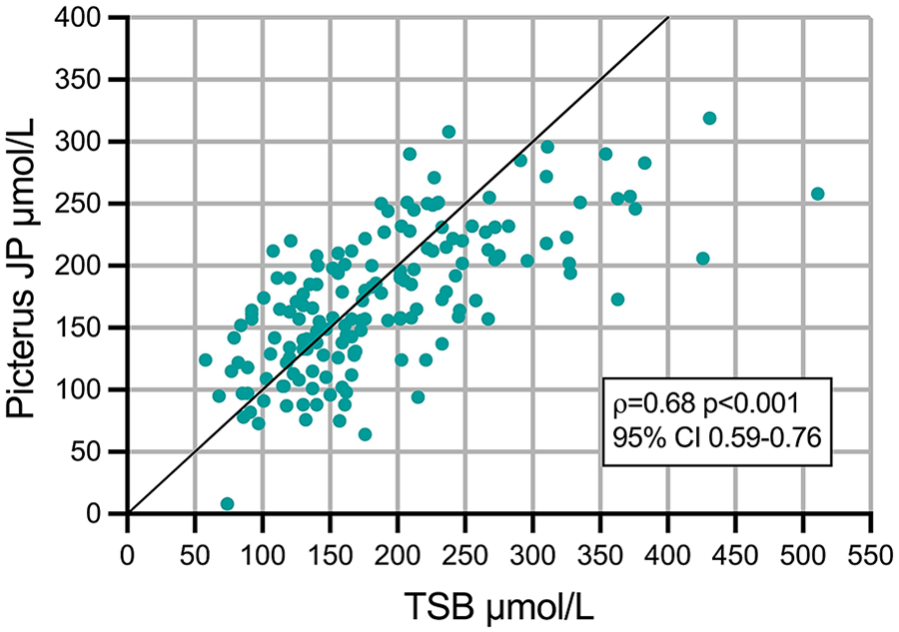
Scatter plot showing the correlation between TSB and Picterus JP. TSB: Total serum bilirubin; μmol/L: Micromoles per litre; ρ: Spearman’s rho Correlation Coefficient; CI: Confidence Intervals. The X-axis represents TSB values, while the Y-axis represents Picterus JP values.

A statistically significant positive correlation was found between TSB and Picterus JP measurements (Spearman’s rho ρ = .68, 95% Confidence interval [CI]: 0.59-0.76, *p* ≤ .001) (Figure 1). There was a significant positive correlation between TSB and the Kramer scale (ρ = .72, 95% CI: 0.63-0.79, *p* < .001).

**Figure 2.**
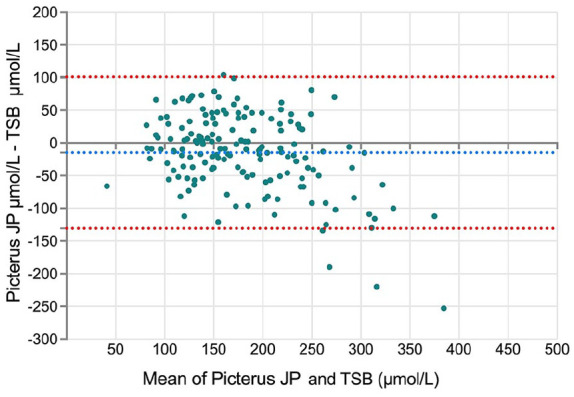
Bland-Altman analysis comparing Picterus JP and TSB values in Micromoles per litre (μmol/L). TSB: total serum bilirubin; SD: standard deviation. The X-axis represents the mean of Picterus JP and TSB values, while the Y-axis shows the difference between Picterus JP and TSB values. The middle-dotted line indicates the mean of the difference, and the top and bottom dotted lines represent the 95% limits of agreement.

A Bland-Altman analysis revealed a mean difference (bias) ± SD of −14.73 ± 59.2 µmol/L (−0.86 ± 3.5 mg/dL) between Picterus JP and TSB, with 95% limits of agreement ranging from −130.7 to 101.25 µmol/L (−7.64 to 5.93 mg/dL) (Figure 2). To further explore the performance of Picterus JP, the dataset was split into two groups based on TSB values: ≤250and >250 µmol/L. Separate Bland-Altman analyses were then performed for each group. For TSB values ≤250 µmol/L, the mean difference was 0.85 ± 45.6 µmol/L, while for TSB values >250 µmol/L, the mean difference was −90.3 ± 59.6 µmol/L (Supplemental Figure 1(a) and (b)). These results indicate that Picterus JP tends to underestimate TSB levels, particularly when values exceed 250 µmol/L.

**Figure 3.**
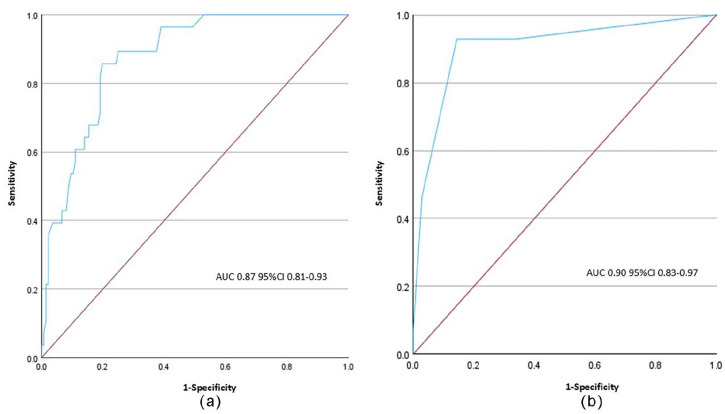
Picterus JP (a) and Kramer scale (b) ROC curve for detecting TSB ≥ 250 μmol/L. μmol/L: Micromoles/Litre; AUC: Area Under the Curve; CI: Confidence Intervals.

ROC analysis to determine Picterus JP’s accuracy in detecting TSB ≥ 250 µmol/L (14.6 mg/dL) showed an AUC of 0.875 (Figure 3(a)). Using a Picterus JP cutoff of 202 µmol/L (11.9 mg/dL) based on the Youden index yielded a sensitivity of 85.7% (correctly identifying most cases), specificity of 80.1% (effectively ruling out cases without severe jaundice), a positive likelihood ratio (+LR) of 4.3, and a negative likelihood ratio (−LR) of 0.17.

Similarly, ROC analysis for the Kramer scale’s accuracy in detecting TSB ≥ 250 µmol/L (14.6 mg/dL) showed an AUC of 0.907 (Figure 3(b)). A Kramer scale cutoff of 3, corresponding to 200 µmol/L (11.7 mg/dL), resulted in a sensitivity of 92.9% and a specificity of 85.6%.

## Discussion

In this study, we evaluated the performance of Picterus JP as a screening tool for NNJ in Mexican newborns at a second-level hospital in a resource-limited setting. Our main findings indicate that Picterus JP has a strong correlation with TSB levels and demonstrates satisfactory accuracy in identifying newborns who may need further diagnosis or follow-up for NNJ. The +LR suggests that a newborn with severe jaundice is 4.3 times more likely to have a positive Picterus JP value (cut-off ≥ 202 μmol/L) compared to one without the condition. However, the device tends to underestimate high bilirubin levels, highlighting areas where improvements are needed. Unlike other reports,^13,34,35^ the Kramer scale showed outstanding performance and accuracy in this study.

The mothers’ skin types, classified using the Fitzpatrick scale, were consistent with the expected population characteristics for this region, where types III to V (light to dark brown) are most common.^36^ However, significant missing data on fathers’ skin types limited our ability to analyse this factor.

The Neomar scale was used to assess newborn skin tones for the first time by the data collection staff, but the results did not align with the expected distribution for the studied population. Nearly 40% of the infants were classified with a score of 1, representing the lightest skin tone, which does not reflect the anticipated variation. This discrepancy highlights the subjective nature of the Neomar scale and its limitations in providing an accurate estimation of actual newborn skin tones. Consequently, these data were not used for further analysis.

Our findings are partially consistent with other studies on smartphone-based technologies designed to estimate bilirubin levels by analysing newborn skin colour. For instance, the BiliCam app, tested on 530 newborns including 26.3% Hispano-Latino, reported a correlation of 0.90 with an overall sensitivity of 100%, specificity of 76%, and a +LR of 4.2 for detecting TSB levels of 291 μmol/L at a cutoff of 222 μmol/L.^16^ The BiliScan app from Shenzhen Beishen Healthcare in China showed sensitivity between 75% and 90% and a TSB correlation ranging from 0.54 to 0.82, mainly in Middle Eastern and Chinese populations. Variations in inclusion parameters and result analysis in BiliScan studies limit the generalizability of their findings.^17,18,37-39^

Our results show a lower correlation between Picterus JP and TSB compared to earlier studies from Norway and Mexico (*r* = 0.85 and 0.87, respectively),^21^ but are consistent with studies from Nepal and the Philippines (*r* = 0.70).^22^ Although the correlation coefficient is commonly used to assess the relationship between two methods, it is not the most suitable for evaluating agreement.^40^ Bland-Altman analysis provides a more appropriate measure by assessing both bias and variability, offering a clearer and more comprehensive picture of test agreement.^41^ Table 4 summarizes the results of this analysis and Picterus JP’s accuracy for detecting TSB ≥ 250 μmol/L across different settings.

**Table 4.** Summary of Results From Studies Using Picterus JP.

Study site	N	Mean difference Picterus JP – TSB		95% limits of agreement		AUC	Sensitivity^#^	Specificity^#^	Picterus JP cut-off
		μmol/L	mg/dL	μmol/L	mg/dL				
Norway^20^	302	−0.2	−0.01	−83.9 to 83.5	−4.9 to 4.9	0.92	87	77	225
Norway^23^	201	–9.7	-0.56	−89.9 to 70.6	−5.6 to 4.1	0.89	94.1	70.7	214
Mexico^21^	166	−6.2	−0.36	−84.0 to 85.2	−4.9 to 5.0	0.92	85.7	85.5	200
Philipinnes^22^	69	15.5	0.9	−60.2 to 91.3	−3.5 to 5.3	0.89	100	75.8	184
Nepal*	182	21.5	1.26	−65.6 to 108.7	−3.8 to 6.3	0.90	88.2	78.8	211
This study	164	−14.7	−0.86	−130.7 to 101.2	−7.6 to 5.9	0.87	85.7	80.1	202

µmol/L: Micromoles per litre; mg/dL: Milligrams per decilitre; AUC: Area Under the Curve.

* Unpublished. ^#^To detect TSB ≥ 250 μmol/L.

This study, consistent with previous research on Mexican newborns, indicates that Picterus JP tends to underestimate TSB, especially for values above 250 μmol/L. This pattern mirrors the underestimation observed with TcB measurements,^8^ possibly due to both Picterus JP and TcB assessing extravascular bilirubin, which can be up to four times lower than blood bilirubin concentration.^42^

Variations in Bland-Altman analysis and accuracy across studies may be attributed to several factors, including differences in image quality, bilirubin kinetics, skin thickness, blood flow, skin maturity, and melanin levels across different races and ethnicities.^43-45^ The use of different prototype versions of the calibration card in previous studies may have led to inconsistent adjustments for lighting conditions across them. Additionally, the diversity in smartphones and Picterus JP versions could contribute to differences. Variations in participant inclusion criteria, study procedures, and laboratory equipment^46^ may also result in discrepancies in TSB values and study outcomes.

However, these factors do not fully account for the wider 95% limits of agreement observed in our study. While these limits suggest that Picterus JP may not be suitable as a standalone diagnostic tool or replacement for TSB tests, it can serve its intended purpose as an effective screening tool. When used with adjusted cut-off values, the device can assist healthcare providers in identifying newborns who require a TSB test for confirmation, thereby reducing unnecessary blood draws and allowing earlier detection of jaundice.

Visual assessment remains a common method for screening NNJ worldwide, but its accuracy and reliability are often questioned due to its subjectivity and dependence on the examiner’s skill.^13,47^ It has generally been more effective at ruling out severe NNJ than detecting it. The outstanding performance of Kramer scale in this study is likely due to the expertise of the paediatric professionals involved in the assessment. These results emphasize the importance of thorough training in visual assessment to improve the identification of severe cases, especially where other diagnostic tools are limited or not available.

### Strengths

Data collection was performed by three trained medical doctors, ensuring high-quality and consistent results. The use of the same laboratory equipment for blood tests minimized potential discrepancies in TSB results.^46^ Although the Asian population was not included in this study, the similarity in skin types between Hispanic-Latino and Asian groups suggests that the device is likely to perform well across these populations, increasing global applicability.

Additionally, as the Mexican government expands mHealth technologies to enhance healthcare services,^48^ the study’s findings support the integration of Picterus JP into the national healthcare system, potentially improving early detection of severe NNJ, especially in resource-limited areas.

### Limitations

The study was conducted in a single general hospital, with 65% of participants being younger than 72 hours. Since bilirubin levels typically continue to rise after the first 72 hours of birth, the results may not fully generalize to older newborns.

Our sample was limited to newborns who required blood tests, avoiding unnecessary invasive procedures. Most of these tests were conducted for routine or minor concerns, such as maternal urinary tract infections, rather than for serious medical conditions. Although the prevalence of severe NNJ (TSB > 250 µmol/L) in our study is consistent with existing estimates for this condition, this sampling limitation should be considered when interpreting the study’s applicability to all newborns.

The selected smartphones were chosen based on availability and reflect commonly used models with different software systems. While the calibration card is designed to correct for variations in camera sensors, we cannot entirely rule out the possibility of bias in the study data due to smartphone variability.

The findings may also be more applicable to newborns with similar skin types to the study population, as Picterus JP’s performance has not been validated for infants with higher melanin content, limiting its generalizability to populations with darker skin tones.

The Kramer scale assessments were performed by highly skilled and experienced professionals in this study. In most healthcare settings, particularly in resource-limited areas, assessments may be conducted by less experienced staff, which could impact the real-world performance of VA methods.

Picterus JP also relies on a stable internet connection, a potential limitation in LMICs where connectivity is often inconsistent. Additionally, the study used smartphones with relatively high-resolution cameras, which may not reflect the technology available in more disadvantaged populations. These factors could limit its applicability in these settings. To ensure broader and equitable access, Picterus JP should be compatible with a wider range of smartphones and function reliably with limited or no internet connectivity. Addressing these challenges will help mitigate digital inequalities and enhance the device’s scalability.

### Future Research

This study underscores the potential of mHealth technologies like Picterus JP to enhance NNJ screening across diverse populations. Key considerations and future directions include:

1. Accuracy enhancement: Refine the image quality algorithm to better handle higher TSB levels.
2. Diverse validation: Test the device on a wider range of skin types, especially those with higher melanin content.
3. Implementation: Evaluate the feasibility of integrating Picterus JP into health systems, focusing on its impact on reducing NNJ-related morbidity and mortality.
4. Accessibility: Reduce reliance on internet connectivity and increase compatibility with various smartphones for better usability in resource-limited settings.
5. Training: Provide comprehensive training for healthcare providers to improve visual assessment accuracy, particularly in settings where other detection tools are limited.

## Conclusions

The study highlights the potential of Picterus JP to support Mexican healthcare workers in identifying newborns at risk of severe NNJ complications. With a significant correlation (ρ = .68) and acceptable accuracy (AUC = 0.875), the device shows potential to be a useful tool for NNJ detection. Appropriate adjustments, such as improving accuracy for high TSB levels and validation in diverse populations, will contribute to enhance Picterus JP’s capability as a screening tool, especially in resource-limited areas. This would contribute to increase early detection and reduce disparities in neonatal health. Continuous investment in research and development, along with collaboration with public health organizations and policy support is essential for wider implementation and scaling up the benefits of mHealth solutions.

## Supplemental Material

sj-docx-1-gph-10.1177_30502225251320544 – Supplemental material for Validation of a Mobile Health Device for Neonatal Jaundice Screening: A Cross-Sectional Study in a Resource-Limited Setting in Mexico Supplemental material, sj-docx-1-gph-10.1177_30502225251320544 for Validation of a Mobile Health Device for Neonatal Jaundice Screening: A Cross-Sectional Study in a Resource-Limited Setting in Mexico by Gabriela Jiménez-Díaz, Lobke M. Gierman, Martina Keitsch, Jesús Elizarrarás-Rivas, Rey Manuel Silva- Méndez, Jennifer J. Infanti and Anna Marcuzzi in Global Pediatric Health
