# Supplementary material for: Validation of a Mobile Health Device for Neonatal Jaundice Screening: A Cross-Sectional Study in a Resource-Limited Setting in Mexico
Source: Sage Open Pediatr. 2025 Apr 22;12:30502225251320544. doi: 10.1177/30502225251320544 (PMC12220874; doi:10.1177/30502225251320544)
Supplement: sj-docx-1-gph-10.1177_30502225251320544 – Supplemental material for Validation of a Mobile Health Device for Neonatal Jaundice Screening: A Cross-Sectional Study in a Resource-Limited Setting in Mexico [file sj-docx-1-gph-10.1177_30502225251320544.docx]

Supplemental Figure 1A and B

*Supplemental Figure 1. Bland-Altman analysis comparing Picterus JP and TSB values ≤250 (A) and >250 (B) Micromoles per litre (µmol/L).*

*TSB: total serum bilirubin; SD: standard deviation.*

*The X-axis represents the mean of Picterus JP and TSB values, while the Y-axis shows the difference between Picterus JP and TSB values. The blue dotted line indicates the mean of the difference, and the red dotted lines represent the 95% limits of agreement.*
